# Factors Related to Curve Progression in Adolescent Idiopathic Scoliosis Girls at Skeletal Maturity

**DOI:** 10.3390/healthcare13222857

**Published:** 2025-11-11

**Authors:** Eddie Geagea, Anna Rambo, Kylie Krombholz, Ali Siddiqui, Kevin M. Neal

**Affiliations:** 1 Department of Orthopedic Surgery, Nemours Children’s Health, Orlando, FL 32827, USA; 2 Department of Orthopedic Surgery, Nemours Children’s Health, Jacksonville, FL 32207, USA; 3 College of Medicine, Florida State University, Tallahassee, FL 32306, USA; 4 College of Medicine, University of Florida, Jacksonville, FL 32611, USA

**Keywords:** adolescent idiopathic scoliosis (AIS), progression, skeletal maturity (SM), cobb angle, risk factor

## Abstract

**Background/Objectives**: 45–50° is typically considered a threshold to offer surgical intervention for adolescent idiopathic scoliosis (AIS) patients. Larger curves may continue to progress even after skeletal maturity (SM), but the risk of this progression remains poorly defined. This study aimed to quantify the risk factors for continued curve progression in skeletally mature females with moderate curves (25° to 45°). **Methods**: We reviewed a Non-Operative AIS Database of >2300 patients with curves of 25° to 45° at radiographic SM (defined as United States Risser 4 and Sanders 7, or Risser 5) and follow-up >24 months after SM. Progression of >5° and progression to 50° were analyzed using chi-squared tests and Mann-Whitney U tests to detect differences in factors at SM. **Results**: 90 patients met the inclusion criteria. For various starting curve sizes at SM, progression > 5° was 7.1% (25°), 26.7% (30°), 22.2% (35°), 42.3% (40°), and 33.3% (45°), respectively. Progression to 50° was 0% (25°), 0% (30°), 11.1% (35°), 42.3% (40°), and 55.6% (45°). Progression > 5° was higher in patients with Risser 4 at SM versus Risser 5 (*p* = 0.04), when curves were >35° at SM (*p* = 0.04), and onset of menarche was <16 months before SM (*p* = 0.03). Progression to 50° was higher for curves > 40° at SM (*p* < 0.00001) and when the onset of menarche was <15 months before SM (*p* = 0.02). **Conclusions**: Curves of 25° to 45° at SM can still progress > 5°, and curves of 35° to 45° can still progress to 50°. Patients should be counseled regarding these risks so they can make informed decisions about appropriate monitoring and treatment.

## 1. Introduction

Scoliosis is defined as a three-dimensional deformity of the spine [1], with idiopathic scoliosis (IS) representing the largest subgroup of spinal curvature in humans, accounting for 70–90% of all known scoliosis cases [2]. IS is characterized by a coronal curvature of the spine, with a measured Cobb angle of at least 10° in the absence of underlying congenital or neuromuscular abnormalities [3]. IS is divided into three subgroups: infantile idiopathic scoliosis (IIS), which develops at 0–3 years of age, juvenile idiopathic scoliosis (JIS), which develops between 4 and 9 years of age, and adolescent idiopathic scoliosis (AIS), which develops at age 10 or greater. AIS accounts for approximately 90% of IS cases, with a prevalence of 1% to 3% of individuals 10 to 16 years of age [4,5]. AIS occurs primarily in girls at a ratio of 1.5:1 (Female:Male) in mild scoliosis and a ratio of up to 10:1 (Female:Male) in scoliosis with a Cobb angle  >  30° [6].

Lonstein and Carlson [7] defined progression as a 5° increase in Cobb angle between two successive radiographs. The same 5° threshold was used in the epidemiologic studies by Brooks et al. [8] and Rogala et al. [9]. Factors associated with progression include skeletal maturity (SM), chronological age, sex, growth potential, and initial curve magnitude [10]. Previous studies have identified age 12 as the average age for menarche [11] and age 15 as the average age for complete cessation of growth in girls [12]. Prior work has highlighted an initial Cobb angle of 25° as an important threshold for long-term curve progression [13]. The Risser sign is the most common skeletal marker used to assess skeletal maturity, and for girls, menarche provides an additional indicator of maturity [14].

Based mainly on long-term natural history data, many surgeons consider 45–50° a threshold above which surgery is recommended, and below which observation is advised [15,16]. Long-term follow-up studies on curve progression prior to SM are comprehensive and have identified various prognostic factors [17,18,19,20,21]. The natural history of untreated AIS after SM has been explored in several case series [15,16,22,23], but data specific to the behavior of curves between 25° and 45° in skeletally mature patients are lacking, as most reports group curves broadly as <30° or >50°.

**Figure 1:**
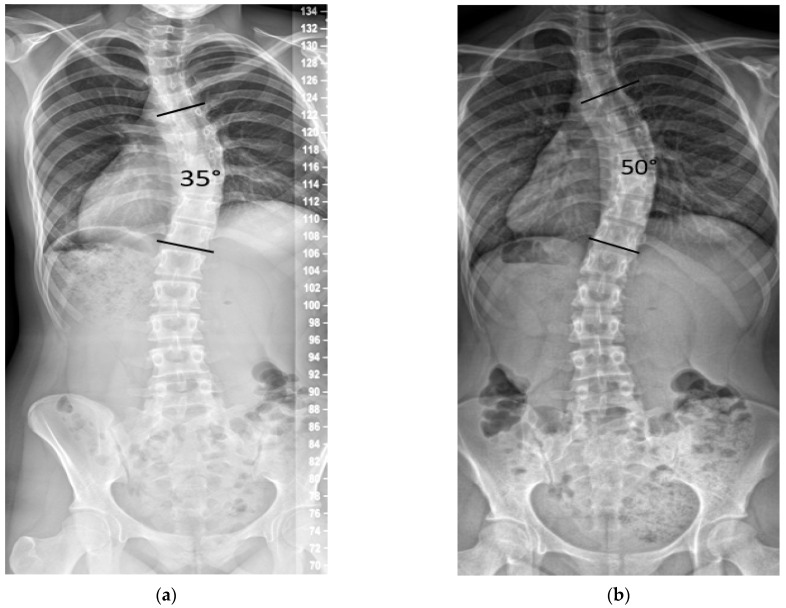
Example of curve progression after skeletal maturity. (**a**) Female age 14.6 years, Risser 5, 28 months post-menarche, height 153.7 cm, main curve 35°. (**b**) Age 17.7 years, height increased by 0.3 cm, and the main curve progressed to 50°.

We noted in our practice that some patients with curves between 25° and 45° progressed after documented SM, with some eventually seeking posterior spinal fusion (PSF) due to dissatisfaction with their spinal deformity (Figure 1).

This study aimed to evaluate the rate of post-maturity curve progression in girls with AIS with Cobb angles between 25° and 45°, and to identify risk factors for short-term curve progression over a minimum follow-up of 24 months.

We hypothesized that among adolescent girls with moderate AIS (25–45°), the risk of post-maturity curve progression increases with larger starting Cobb angle, lower Risser stage (4 vs. 5), shorter intervals since menarche, greater height velocity, and thoracolumbar/lumbar (vs. thoracic) curve location.

## 2. Materials and Methods

Institutional Review Board (IRB) approval was obtained from our pediatric tertiary hospital to conduct a retrospective cohort study using a prospectively collected non-operative AIS database, which includes radiographic and demographic data for more than 2300 female patients.

A grading system for ossification of the iliac apophysis, known as the Risser sign, was developed to assess skeletal maturity, the US Risser stage uses a scale from 0 to 5, where stage 5 indicates complete ossification and SM [24,25]. The European Risser stage also uses a scale from 0 to 5, but the criteria for each stage differ slightly. For instance, in the European system, stage 4 indicates complete ossification, which corresponds to stage 5 in the U.S. system [24,25]. The differences in grading can lead to discrepancies in treatment decisions. For example, the U.S. system tends to assign a higher Risser grade compared to the European system for the same level of ossification, which can influence the timing of interventions such as bracing or surgery [25]. The US Risser stage is considered more effective for determining the maturity of girls with AIS, especially when combined with the number of years since menarche, thereby enhancing the accuracy of growth cessation predictions [24]. The Sanders system classifies SM on anteroposterior hand-wrist radiographs, using epiphyseal capping and stepwise physeal closure across the phalanges and metacarpals to assign eight stages (1–8). Stage 3 coincides with the curve-acceleration/peak-height-velocity period when curves increase most rapidly. The risk of significant progression declines across later stages (4–8) as physes close. Stage 7, where all phalangeal physes are closed, has been suggested as a marker of relative SM with minimal growth remaining, and Stage 8 represents absolute SM with the distal radial and ulnar physes completely closed [26]. Inclusion criteria were: (a) a diagnosis of adolescent idiopathic scoliosis (AIS); (b) skeletal maturity (SM) at the index visit, defined as United States (US) Risser 5 or US Risser 4 AND Sanders stage 7 or 8, (c) a curve Cobb angle of 25–45° at the SM visit; and (d) at least 24 months of follow-up after SM, with no bracing or other therapeutic interventions. Exclusion criteria were: non-idiopathic scoliosis; prior spine surgery at or before the SM visit; not meeting criteria for maturity at the initial visit, no follow-up for a minimum of 24 months after maturity; and incomplete radiographic data. If patients progressed by >5° or to 50° before the 24-month follow-up, they were still included. For patients who underwent surgery, the last preoperative Cobb angle was used as the final measurement.

All patients underwent standing posteroanterior (PA) scoliosis radiographs at the SM visit and at follow-up visits. All Cobb angles, Risser, and Sanders scores were measured and entered into the database by the treating fellowship-trained pediatric orthopedic surgeon.

Because Cobb measurements show intra- and interobserver variability of 4–8°, values were rounded to the nearest 5° to reduce the influence of small measurement variability, to simplify analysis, and to remain consistent with prior studies of the natural history of AIS [26,27,28]. (Recorded 25° curve = 23° to 27°. Recorded 30° curve = 28° to 32°. Recorded 35° curve = 33° to 37°. Recorded 40° curve = 38° to 42°. Recorded 45° curve = 43° to 47°).

Curve progression was evaluated in two ways: (1) any progression > 5° and (2) progression to 50°. Categorical outcomes (progression > 5° and progression to ≥50°) were compared between groups using Chi-square tests, or Fisher’s exact test when any expected cell count was <5° A post hoc power analysis showed that our power to detect a difference between the 30° and 40° subgroups for 10° of progression at these sample sizes (19 and 22 patients) was only 45%. Sixty-five patients would be required for 90% power for subgroup comparisons, which will likely require several more years of data collection. No sample size calculation was performed because this was a convenience sample.

Additional variables analyzed included age at SM, duration of follow-up, height velocity between initial and final visits, Risser stage at SM, Sanders stage at SM, curve magnitude at SM, main location of the primary curve thoracic vs. thoracolumbar/lumbar (TL/L), and time since menarche (in months) at SM. Continuous variables were analyzed with Mann-Whitney U tests to detect differences in factors that might influence curve progression. All statistical analyses were performed using publicly available statistical calculators from www.socscistatistics.com.

## 3. Results

Ninety patients met the inclusion criteria for the study, with an average follow-up length of 37.5 months. At initial presentation, 60% had a thoracic curve (54/90) and 40% had a thoracolumbar or lumbar (TL/L) curve (36/90). The distribution of initial curve magnitude was 25° in 16% of patients, 30° in 17%, 35° in 30%, 40° in 26%, and 45° in 9%.

At the time SM was first documented, the mean age was 13.0 years old, and the majority of patients (79%, 71/90) had a Risser score of 4. On average, SM was recorded 19 months following the onset of menarche.

The frequency of progression > 5° from starting curves (25–45°) was seen in 7.1% (25°), 26.7% (30°), 22.2% (35°), 42.3% (40°), and 33.3% (45°), respectively. The frequency of progression to a curve ≥50° was seen in 0% (25°), 0% (30°), 11.1% (35°), 42.3% (40°), and 55.6% (45°) (Table 1).

Progression > 5° (*n* = 25) was statistically significant when patients were Risser 4 at SM versus Risser 5 (*p* = 0.04), had curve magnitude > 35° at SM (*p* = 0.04), or if the onset of menarche was <16 months before SM (*p* = 0.03). Chronologic age at SM, length of follow-up, height velocity, SMS at SM, and curve location were not predictive of curve progression > 5° (All *p* > 0.05) (Table 2). 

Progression to 50° (*n* = 19) was significantly associated with a curve magnitude >40° at SM (*p* < 0.00001) and onset of menarche <15 months before SM (*p* = 0.02). Chronologic age at SM, length of follow-up, height velocity, Risser stage at SM, SMS at SM, and curve location were not predictive of curve progression ≥50° (All *p* > 0.05) (Table 3).

## 4. Discussion

Accurate prediction of curve progression in patients with AIS remains critical, as it is the primary indication for surgical intervention. However, it remains a challenging task, as clinical decisions depend on identifying a reliable marker that indicates the cessation of curve progression [29]. AIS curves between 25° and 45° in females after SM represent a controversial therapeutic zone. Understanding their natural history is important, as we found that even after SM, curves ≥25° carried a measurable risk of progression, with some curves ≥35° at SM reaching the surgical threshold of ≥50° within a relatively short follow-up period.

While surgery for AIS can be considered elective, and progression does not appear to reduce life span or significantly limit activity [30], patients with larger curves who choose observation after SM may later choose surgical intervention to prevent further deformity, particularly for cosmetic concerns. Scoliosis Research Society (SRS) questionnaire domains for pain and function are typically not altered after surgical intervention [18]. Surgery is typically offered as an option for patients with larger curves that are cosmetically concerning and that have some risk of progressing. However, the exact magnitude at which progression becomes inevitable is not known.

Our findings indicate that curves measuring 25–45° at SM, particularly those ≥35°, carry a measurable risk of progression, both in terms of exceeding 5° and reaching the surgical threshold of ≥50°. Progression >5° occurred in up to 42.3% of patients with 40° curves and 33.3% of those with 45° curves. Notably, 42.3% and 55.6% of patients in these respective groups progressed to a surgical threshold of ≥50°. Because 45° curves are already close to 50°, some could have reached ≥50° with <5° change. Some patients likely chose surgical intervention at 45°, decreasing the number of patients available for analysis. In contrast, no patients chose surgical intervention at 40°, leaving a larger pool for analysis of progression >5°. These findings indicate that SM does not guarantee curve stabilization over the relatively short follow-up period of this study. The likelihood of progression increased with the magnitude of the curve at maturity, supporting the conclusion of Tan KJ et al. [31] that the initial Cobb angle is the most important predictor of long-term curve progression and behavior past SM; our results also align with their findings that initial age, gender, and pubertal status were less critical prognostic factors. By contrast, none of the patients with baseline curves of 25° or 30° reached the surgical threshold. These findings are consistent with previous reports, such as Weinstein et al. [16], which observed that curves ≥30° at SM are prone to adult progression, while smaller curves <30° generally remain stable. However, their study included very few patients in the 30–45° range at SM, while our study extends previous knowledge by quantifying progression risk specifically within the 25–45° range, thereby allowing more granular risk estimates for this intermediate group.

Historical data from Collis and Ponsetti were published in the first long-term natural history study of scoliosis from Iowa in 1969 [32]. They described the natural history of IS in 134 patients who were followed for an average of 24 years. They reported on 119 patients at Risser stages 4 and 5. Of those, 84 (71%) progressed more than 5 degrees by final follow-up, and 38 (32%) progressed more than 15 degrees. This is consistent with our results, that some 30–40° curves can progress after SM. However, their lack of curve size specification at maturity limits direct comparison. Similarly, Scott and Piggott [22] observed progression in 70% of patients post-maturity, but only 11 of the curves were between 30° and 40° at SM, and their results showed less progression than ours. Of those 11 patients, 8 (73%) progressed 5° or more over an average follow-up of nearly 11 years, but none progressed ≥10°. Bjerkreim et al. [33] also confirmed greater progression in larger curves, though their reporting did not permit specific analysis of 30–40° curves. Weinstein et al. [15,16,30] included only a few patients in the 30–40° range but found comparable progression rates.

In the Iowa study published in 1981, there were a total of 24 patients with curves between 30° and 40° at SM [15]. Of those, 17 (71%) appeared to have documented progression as of their latest follow-up. In a similar study, published in 1983 with an average follow-up of 40.5 years, 17 patients had a curve between 30° and 40°, and 8 (47%) appeared to progress [16]. However, the Weinstein studies rely on graphs showing starting points and subsequent progression, rather than detailing the exact degrees for each. Their data also suggested a higher risk of progression for lumbar and thoracolumbar curves after SM, compared to thoracic curves. Our study did not identify curve location as a significant predictor of progression. Specifically, our findings showed that thoracolumbar/lumbar (TL/L) curves exhibited lower rates of progression compared to other curve types after SM, despite comprising 40% of the cohort. Although the difference was not statistically significant, the trend aligns with Ascani et al. [34], who similarly reported less progression in isolated lumbar and thoracolumbar curves over long-term follow-up. For thoracic curves between 30° and 39°, they noted an average progression of about 11°, and for the lumbar component of double major curves, 12° to 14° of progression. These consistent findings suggest that TL/L curves may carry a lower risk of progression after SM, though larger subgroup analyses are needed to confirm this pattern.

A recent study by Grothaus et al. [29] also documented that among AIS patients at Sanders 7 with curves between 40° and 50°, 58% progressed beyond 50° or had surgery. This underscores substantial late progression risk, which mirrors our finding of high risk of progression in 40–50° curves after SM.

Our significant risk factors were lower Risser stage 4, and initial curve magnitude > 35° for general progression or >40° for reaching 50°. Post-SM height velocity in our cohort was minimal and not different between outcomes, averaging 0.5 cm/year in patients whose final curve remained <50° vs. 0.4 cm/year in those reaching ≥50°; *p* = 0.22, yet progression was observed in both groups. This observation aligns with Shitozawa et al. [35], who showed that even at late maturity, height velocity is very low, Risser 4 ~0.9 cm/year, yet curve changes could still be observed. These findings are also consistent with the systematic review by Lenz et al. [6], which identified initial curve magnitude and SM as the most relevant predictors and discusses the limitations of relying on Risser stage alone, suggesting that radiographic SM may not fully capture biological maturity or residual growth potential. Prior analytical biomechanical modeling studies have demonstrated that once a spinal curvature is present, asymmetrical spinal loading drives asymmetrical vertebral growth, perpetuating a self-reinforcing “vicious cycle” of deformity progression despite minimal remaining stature gain [36].

Another significant factor for curve progression was a shorter time between menarche and SM (<15–16 months). Existing literature demonstrates that menarche status is a predictive factor for curve progression. Height velocity, which corresponds to the curve acceleration phase of growth, is fastest in the 6–12 months prior to menarche [37]. Curve progression slows after menarche, becoming negligible beyond 2 years [38]. This supports our finding that a higher risk of progression was observed when the onset of menarche occurred less than 15 months before SM. In a previous study, Neal et al. also discussed the importance of menarche as a factor to consider when assessing SM [37].

Although Nam et al. [10] have identified chronological age as a significant predictor of curve progression, these analyses included patients at varying stages of SM. In contrast, our study focused exclusively on girls who had already reached SM, a stage at which spinal growth has largely ceased. As a result, variability in growth potential is minimal, which may explain why age was not a significant factor in our cohort.

In this cohort, a larger Cobb angle at SM predicted progression (>5° and ≥50°), and Risser 4 (vs. 5) was associated with >5° progression but not with reaching ≥50°. A shorter interval since menarche correlated with both endpoints. In contrast, residual height velocity showed no association with either outcome, and curve location (TL/L vs. thoracic) was not predictive at either level. Overall, the hypothesis was only partially supported.

During follow-up, all patients in our cohort were managed with observation only. In our practice, the standard treatments for AIS include observation, bracing, or surgery. The use of scoliosis-specific exercises (SSE) for AIS remains controversial. While SSE has been routinely used in several Mediterranean and continental European countries, broader uptake elsewhere has been limited [39]. At our institution, SSE is not part of the typical scoliosis treatment protocol.

By definition, the post-SM patients (Risser 4–5) did not meet bracing criteria. Our institution uses the criteria defined by Cheung et al. [40] that specify brace initiation when patients are Risser 0–2 and <1-year post-menarche. There is no consensus on how long patients should be braced or whether braces should be discontinued once reaching some objective versus a weaning protocol [41]. At our institution, braces are discontinued without weaning when patients are at least Risser 4, Sanders 7, and show decreased height velocity to <2.5 cm/year. Roye et al. [42] state that a skeletally mature patient (Risser 5, Sanders 7 or 8, minimal to no growth in 1 year) is not a brace candidate (Grade A consensus recommendation; consensus = ≥80% agreement). Although U.S. Risser is commonly used as a maturity marker in clinical practice, our cohort shows that patients at U.S. Risser 4 at the SM visit were more likely to experience > 5° progression than those at Risser 5; therefore, Risser 4 should not be assumed to be fully mature. At the same time, Risser’s stage did not distinguish who would reach ≥50°. Two factors were more informative across endpoints: a larger Cobb angle at SM (>35° for >5°; ≥40° for ≥50°) and a shorter interval since menarche (<16–15 months, respectively). In our dataset, Sanders stage 7 versus 8 at SM was not predictive for either endpoint, likely reflecting limited availability, yet consistent with Cheung et al. [40], bone-age measures such as the Sanders stage offer finer resolution near the maturity boundary and provide clearer brace-weaning thresholds (e.g., protection at Sanders 8) when residual growth is most consequential. Consensus statements from Negrini et al. emphasize that U.S. Risser 4 remains within bracing candidacy, whereas Risser 5 generally does not, with decisions being individualized based on curve magnitude, maturity staging, and patient goals [43].

The observed association between a shorter post-menarche interval, a lower Riser score, and progression in our cohort suggests that these factors might be included in the decision to continue bracing longer.

Thoracic deformity, particularly rib cage deformity, may be a crucial driver of curve behavior in AIS. Shea et al., evaluated thoracic deformity parameters to predict progression in skeletally mature AIS curves of 40–50°, reporting that modified Nash-Moe apical axial rotation and the Rib Index (measured on thoracic curves at maturity) each predicted accelerated deterioration, and that a combined model using both measures performed best [44,45]. A recent review by Grivas et al. [45] highlights the importance of the thorax in scoliotic deformity and introduces two factors that describe the deformity of the rib cage, the Rib Index and the Segmental Rib Index, as predictors of IS progression. Although our cohort centers on 25–45° curves (somewhat smaller than the 40–50° cohort studied by Shea et al.), the same thoracic-deformity mechanisms could be relevant. However, our study did not include rib cage measures. Additionally, rib prominence measurements may be harder to accurately ascertain in curvatures < 40° [44], and were also not obtained in our cohort.

Our study has some limitations; In addition to its retrospective design, our follow-up averaged 25 months, so we were unable to demonstrate whether the curves had continued to progress or stabilized later in life. Our practice treats pediatric patients exclusively, so it is rare for us to have data beyond age 18 years. As such, the results of this study should be interpreted within the context of a relatively young adult population. Bjerkreim et al. found that rates of progression were lower in patients over 20 years old with curves up to 60 degrees when compared to patients aged 16 to 20 years with similar curve sizes [33]. We initially analyzed patients with starting points of 45°, but many of those with larger curves (45–50°) opted for surgery, limiting our ability to observe the natural history beyond that point. Our study analyzed curve progression in relation to curve size, age, menarche, Risser, SMS, and brace treatment, but did not analyze other factors such as BMI/body composition, tissue laxity, markers of bone health, rib cage deformity, or family history. Prior work has linked family history and low bone mineral density to a greater risk of progression [6], and AIS patients are often leaner with reduced muscle and fat mass [46]. These factors could affect menarche timing, axial loading, and bone turnover, and may confound our observed associations with a larger Cobb angle at SM, Risser 4, and a shorter post-menarche interval. Future studies could measure and adjust for these variables. Our study primarily relied on Risser 4 and 5 as the definition for SM. We also included 12 months post-menarche as part of our definition of maturity, but both menarche and Risser are known to be less accurate than SMS staging [47]. We only had SMS staging available for 54% of our patients. Finally, all measurements were performed once by a single surgeon, limiting our ability to control for Cobb angle measurement error. Future studies with multicenter collaboration could enroll larger patient cohorts, extend follow-up, and incorporate more comprehensive assessments of clinical and demographic factors to enhance generalizability.

## 5. Conclusions

This study offers one of the few focused analyses on the natural history of moderate (25–45°) AIS curves in skeletally mature girls with at least 24 months of follow-up. Curves measuring 25–45° can still progress, particularly those ≥35°, which showed a high risk of surpassing 40° and reaching the standard surgical threshold of ≥50° within 24 months. At our institution, curves > 40° are considered for surgery in conjunction with shared decision-making involving patients and their families. Risser 4 should not be assumed to have full maturity and can still progress; additional factors, such as Sanders staging and a shorter interval since menarche, can help guide decisions about continued monitoring or intervention. Progression was significantly associated with Risser stage 4 and a shorter interval between menarche and SM. Although some curves ≤ 35° progressed > 5°, none reached 50°, identifying this subgroup as lower risk. This information can help provide accurate curve counseling for patients with AIS and their families regarding future expectations and treatment options. Our results suggest that clinical follow-up after SM to rule out further progression is indicated for curves >30°.

## Figures and Tables

**Table 1:** Curve progression after SM1 by starting curve magnitude. Values are percentages of patients with progression > 5° or ≥50°.

Starting Curve Magnitude at SM	Progression > 5° (%)	Progression ≥ 50° (%)
25°	7.1	0.0
30°	26.7	0.0
35°	22.2	11.1
40°	42.3	42.3
45°	33.3	55.6

SM, skeletal maturity.

**Table 2:** Comparison of clinical and radiographic characteristics at SM between patients with and without curve progression after SM. Curve progression is categorized as ≤5° vs. >5°.

Variable	<5° (*n* = 65)	>5° (*n* = 25)	*p*-Value
Age (yrs)	13.6	13.2	0.08
Follow-up length (months)	37.2	38.5	0.79
Height Velocity (cm/yr)	0.5	0.5	0.44
Starting Risser 4 (%)	72.3	92.0	0.04 *
Starting Sanders 7 (%)	85.5	95.8	0.18
Starting Curve Size (°)	34.2	37.2	0.04 *
Curve location TL/L (%)	40.0	32.0	0.48
Post-menarche at SM (months)	22.8	15.9	0.03 *

* indicate statistical significance between groups (*p*-value ≤ 0.05); TL/L, thoracolumbar/lumbar curve; SM, skeletal maturity.

**Table 3:** Comparison of clinical and radiographic characteristics at SM between patients with and without curve progression after SM. Curve progression is categorized as <50° vs. ≥50° final Cobb angle.

Variable	<50° (*n* = 71)	≥50° (*n* = 19)	*p*-Value
Age (yrs)	13.6	13.2	0.15
Follow-up length (months)	37.6	37.4	0.94
Height Velocity (cm/yr)	0.5	0.4	0.22
Starting Risser 4 (%)	74.6	89.5	0.17
Starting Sanders 7 (%)	86.9	94.4	0.38
Starting Curve Size (°)	33.5	40.5	0.00001 *
Curve location TL/L (%)	39.4	31.6	0.53
Post-menarche at SM (months)	22.6	14.5	0.02 *

* *p*-values indicate statistical significance between groups (*p*-value ≤ 0.05); TL/L, thoracolumbar/lumbar curve; SM, skeletal maturity.

## Data Availability

The data presented in this study are available on request from the corresponding author. The data are not publicly available due to privacy or ethical restrictions.
